# Trofinetide for Rett Syndrome: Highlights on the Development and Related Inventions of the First USFDA-Approved Treatment for Rare Pediatric Unmet Medical Need

**DOI:** 10.3390/jcm12155114

**Published:** 2023-08-04

**Authors:** Shuaibu A. Hudu, Fayig Elmigdadi, Aiman Al Qtaitat, Mazen Almehmadi, Ahad Amer Alsaiari, Mamdouh Allahyani, Abdulelah Aljuaid, Magdi Salih, Adel Alghamdi, Mohammad A. Alrofaidi, Abida, Mohd Imran

**Affiliations:** 1 Department of Basic Medical and Dental Sciences, Faculty of Dentistry, Zarqa University, Zarqa 13110, Jordan; 2 Department of Anatomy and Histology, Faculty of Medicine, Mutah University, Karak 61710, Jordan; 3 Department of Clinical Laboratory Sciences, College of Applied Medical Sciences, Taif University, P.O. Box 11099, Taif 21944, Saudi Arabia; 4 Department of Pharmaceutical Chemistry, Faculty of Clinical Pharmacy, Al-Baha University, P.O. Box 1988, Al-Baha 65779, Saudi Arabia; 5 Department of Pharmaceutical Chemistry, Faculty of Pharmacy, Northern Border University, Rafha 91911, Saudi Arabia; aqua_abkhan@yahoo.com

**Keywords:** Trofinetide, Daybue, NNZ-2566, Rett syndrome, rare diseases, development, patent, prospects

## Abstract

Rett syndrome (RTT) is a rare disability causing female-oriented pediatric neurodevelopmental unmet medical need. RTT was recognized in 1966. However, over the past 56 years, the United States Food and Drug Administration (USFDA) has authorized no effective treatment for RTT. Recently, Trofinetide was approved by the USFDA on 10 March 2023 as the first RTT treatment. This article underlines the pharmaceutical advancement, patent literature, and prospects of Trofinetide. The data for this study were gathered from the PubMed database, authentic websites (Acadia Pharmaceuticals, Neuren Pharmaceuticals, and USFDA), and free patent databases. Trofinetide was first disclosed by Neuren Pharmaceuticals in 2000 as a methyl group containing analog of the naturally occurring neuroprotective tripeptide called glycine-proline-glutamate (GPE). The joint efforts of Acadia Pharmaceuticals and Neuren Pharmaceuticals have developed Trofinetide. The mechanism of action of Trofinetide is not yet well established. However, it is supposed to improve neuronal morphology and synaptic functioning. The patent literature revealed a handful of inventions related to Trofinetide, providing excellent and unexplored broad research possibilities with Trofinetide. The development of innovative Trofinetide-based molecules, combinations of Trofinetide, patient-compliant drug formulations, and precise MECP2-mutation-related personalized medicines are foreseeable. Trofinetide is in clinical trials for some neurodevelopmental disorders (NDDs), including treating Fragile X syndrome (FXS). It is expected that Trofinetide may be approved for treating FXS in the future. The USFDA-approval of Trofinetide is one of the important milestones for RTT therapy and is the beginning of a new era for the therapy of RTT, FXS, autism spectrum disorder (ASD), brain injury, stroke, and other NDDs.

## 1. Introduction

**Figure 1:**
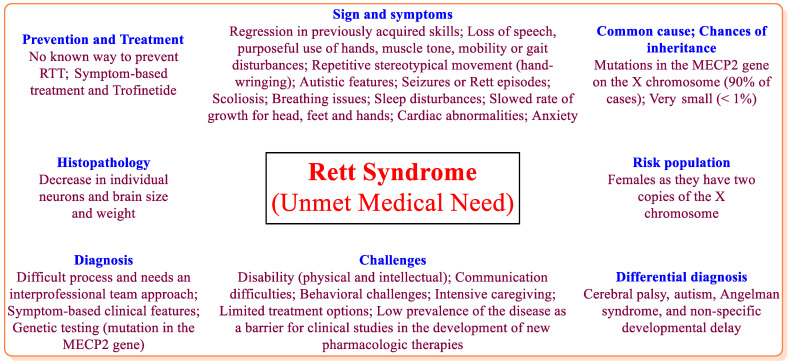
General aspects of RTT [9,10,11,12,13].

Rett syndrome (RTT), a rare disability causing neurodevelopmental syndrome and an unmet medical need, was first characterized by Andreas Rett in 1966 [1,2]. However, this disorder was first officially recognized in a publication form in 1983 by Bengt Hagberg [3]. In 1999, mutations in the methyl-CpG binding protein 2 (MECP2) gene on the X chromosome was found to be the primary cause of RTT by Zoghbi [2,4,5]. Studies have also highlighted the involvement of forkhead box G1 (FOXG1) and cyclin-dependent kinase-like 5 (CDKL5) genes in some cases of RTT [6]. However, the mutation in the MECP2 gene is found in >90% of the RTT cases. RTT is a female-oriented genetic disorder (X-chromosome-linked disorder) that affects mainly girls and young women at a rate of about 1 in 10,000 to 15,000 births, wherein about 10% of females with significant intellectual disabilities have RTT [1,7,8]. RTT causes profound disabilities that permeate practically every facet of a child’s life (the ability to speak, walk, eat, and even breathe). The sign and symptoms of typical RTT (>95% cases) are commonly diagnosed between the ages of 6 and 18 months, whereas the atypical RTT symptoms may appear before the age of 6 months. Between one and four years of age, RTT patients lose the ability to perform skills (loss of language, communication, motor control, walking, social interaction, etc.) that they previously had and develop stereotypies and ataxia. The loss of communication skills in RTT patients is considered the top concern among caregivers. Later in the disease, patients may develop muscle weakness, joint contractures, scoliosis, and seizures. RTT also affects cholesterol metabolism, general growth, and gastrointestinal and renal systems and may cause other diseases (osteoporosis, aspiration pneumonia, pressure ulcers, and infectious disorders). The life expectancy of an RTT patient is approximately 25 to 50 years of age (Figure 1) [9,10,11,12,13].

RTT is a disability-causing disease that can cause lasting neurological damage or even death in young children. The difficulty in diagnosing RTT, the prevalence of different diseases associated with RTT, the availability of limited treatment options, and high healthcare costs make it a significant unmet medical need [14,15,16]. Therefore, effective treatments for RTT are urgently needed in the clinic. Until 10 March 2023, there were no FDA-approved therapies for RTT, and most treatment options for people with RTT focused on relieving the syndrome’s symptoms and effects (multidisciplinary and interprofessional approach). The USFDA approved Trofinetide as the first RTT treatment on 10 March 2023 (Table 1) [17,18,19,20,21].

This article aims to spotlight the pharmaceutical development, patent literature, and prospects of Trofinetide. The data for this study were gathered from PubMed (Trofinetide = 15 hits; Daybue = 15 hits; NNZ-2566 = 10 hits), authentic websites (Acadia Pharmaceuticals, Neuren Pharmaceuticals, USFDA, International Rett Syndrome Foundation, Clinicaltrial.gov, PubChem, etc.), and free patent databases such as the United States Patent and Trademark Office (USPTO) (Trofinetide = 13 hits; Daybue = 0 hits; NNZ-2566 = 42 hits) and Espacenet (Trofinetide = 19 hits; Daybue = 0 hit; NNZ-2566 = 50 hits) utilizing different keywords. The duplicate articles were removed, and articles/authentic websites providing relevant information about the subject matter were included in this article. This article’s scientific content will help pharmaceutical, academic, and healthcare scientists plan further Trofinetide-based research and discover better treatments for NDDs, including RTT, FXS, autism spectrum disorder (ASD), brain injury, stroke, and other NDDs.

## 2. Trofinetide

### 2.1. General Information

**Figure 2:**
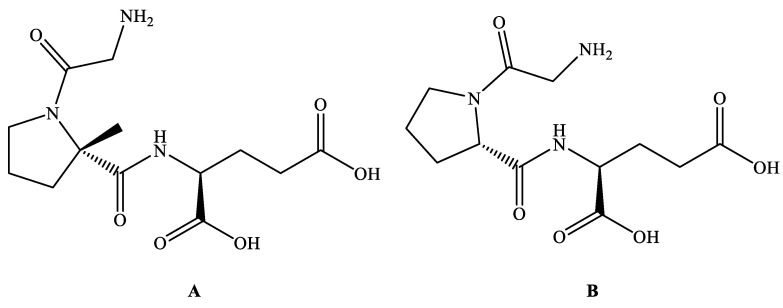
Chemical structure of Trofinetide (**A**) and GPE (**B**).

Trofinetide (Figure 2A; Synonyms: Daybue, NNZ-2566, Glycyl-L-2-methylprolyl-L-glutamic acid and G-2-MePE; MF: C_13_H_21_N_3_O_6_; MW: 315.33; CAS Number: 853400-76-7; Chemical name: (2S)-2-{[(2S)-1-(2-aminoacetyl)-2-methylpyrrolidine-2-carbonyl]amino}pentanedioic acid and Glycyl-L-2-methylprolyl-L-glutamic acid; Class: Peptide) is a peptidase-resistant analog of a naturally occurring neuroprotective tripeptide called glycine-proline-glutamate (GPE) (Figure 2B), which is a N-terminal tripeptide product of the cleavage of insulin-like growth factor 1 (IGF-1) found in the brain [14,22,23,24,25]. Neuren Pharmaceuticals initially identified Trofinetide, which was later developed by Acadia Pharmaceuticals for treating RTT [26].

Trofinetide is soluble in water and can cross the blood–brain barrier. Daybue (shelf life = 18 months at 5 °C) is ready-to-use marketed pink-to-red solution (200 mg/mL) of Trofinetide (Table 1) containing other inactive ingredients (purified water, strawberry flavor, FD&C Red No. 40, sucralose, maltitol, propylparaben sodium, and methylparaben sodium) [22,23].

### 2.2. Mechanism of Action

**Figure 3:**
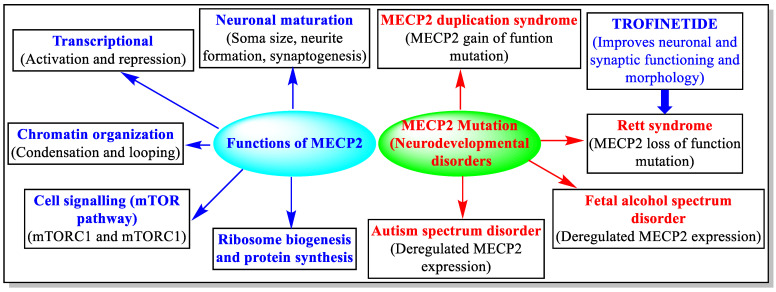
Functions and disorders associated with MECP2 mutations.

The X-linked MECP2 gene produces the MECP2 protein. The MECP2 protein is responsible for various important processes for normal body functions, including brain development and functions. The mutations in the MECP2 gene produce faulty MECP2, affect normal brain development and its function, and cause various disorders, including RTT (Figure 3) [2,5,27,28]. There are about 900 mutations reported in MECP2 genes, most of which arise naturally. To date, the precise means of the pathogenesis of RTT or the exact mutation responsible for RTT is unclear. Studies have suggested various mechanisms of the pathogenesis of RTT via MECP2 gene mutations [12]. One suggested mechanism is that the MECP2 gene mutation retards the normal developments of neurons, axodendritic connections, and the cortex’s synaptic maturation [12]. Another suggested reason for developing RTT is related to the overexpression of IGF-binding protein 3 (IGFBP3) in the brain of RTT patients. It is believed that the MECP2 gene directly regulates the expression of IGFBP3, and a mutation in MECP2 genes can lead to the development of RTT [29].

Trofinetide is the first USFDA-approved treatment for RTT. The specific method by which Trofinetide exercises its effect to treat RTT is uncertain. There are many studies indicating that Trofinetide exerts its effect by improving synaptic functions, restoring synaptic structure, reducing the effects of neuro-inflammatory substances in the brain, enhancing antioxidant responses, attenuating injury-induced apoptosis, normalizing the synthesis of essential proteins, restoring normal homeostasis in the brain, and augmenting the concentration of IGF-1 in the CNS [8,24,26,30,31,32,33,34]. According to the sponsor (Acadia Pharmaceuticals and Neuren Pharmaceuticals), Trofinetide improves neuronal and synaptic functioning (synaptic plasticity signals) and morphology (increased branching of dendrites) [35,36,37,38]. Trofinetide is a methyl analog of GPE (Figure 2). At nanomolar doses, GPE protects neurons from excitotoxicity and oxidative stress caused by glutamate. Trofinetide is thought to have similar neuroprotective characteristics (reduces apoptosis, infarct size, inflammation, and excitotoxicity-induced tissue damage to protect neurons) to GPE but with a longer half-life [26].

### 2.3. Pre-Clinical Studies

Animal studies related to Trofinetide, including its neuroprotective and anti-inflammatory effects in rat brains, are provided in the literature [24,26,30,32]. The USFDA’s non-clinical review document provides non-clinical study data on Trofinetide [36,37]. A short description of the important non-clinical study findings of Trofinetide from USFDA’S documents is provided in Table 2 [36,37].

### 2.4. Clinical Studies

We searched RTT-based clinical studies on the clinical trial database for Trofinetide utilizing the terms “Trofinetide”, “Daybue”, and “NNZ-2566” [39]. Table 3 summarizes the findings of this search.

Our PubMed search revealed the following relevant clinical studies on Trofinetide.

One Phase I study evaluated the influence of food and the evening oral dosing of Trofinetide (60 mL solution, 12 g Trofinetide) on its pharmacokinetic behavior [40]. This study in healthy individuals demonstrated that the bioavailability of Trofinetide was unaffected by diet, showed no diurnal change, and was primarily excreted through urine.

The data of the Phase II study (NCT02715115) have been publicized [8]. Trofinetide (200 mg/kg bid) demonstrated clinical benefits (safety, efficacy, and tolerability) in RTT patients. Another Phase II study also revealed the clinical benefits of Trofinetide (safety, efficacy, and tolerability) at 35 to 70 mg/kg doses [34].

The design and outcome measures of NCT04181723 have been published [14] without results.

A clinical study characterized the population pharmacokinetics of Trofinetide (6–100 mg/kg, oral) among 60 healthy subjects (19–38 years). Trofinetide had a predicted clearance of 10.35 L/h and a center volume of distribution of 20.23 L in the population. There was no treatment-related accumulation, metabolic inhibition, or induction. Because of its short half-life (1.4 h), Trofinetide was suggested to be administered twice or thrice daily [41].

Detailed RTT-based clinical study data of Trofinetide are also provided in the USFDA’s published document [35.42], including the two Phase III clinical studies (NCT04181723 and NCT04279314). The Phase III clinical trial data are utilized to make a drug product’s labeling instructions/information [38]. The important labeling instructions/information of Trofinetide have been discussed in this manuscript’s pharmacology part (Section 2.5). Therefore, to avoid redundancy, the authors did not find it worthy of elaborating on the outcomes of the clinical Phase III data of Trofinetide.

### 2.5. Pharmacology

The important pharmacological aspects of Trofinetide mentioned in the USFDA’s documents are summarized in Table 4 [22,23,35,36,37,38,39,42].

## 3. Patents Literature

Trofinetide was explored on the free patent databases, including Espacenet and USPTO [43,44,45,46], on 6 May 2023, utilizing different keywords (Trofinetide; Daybue; NNZ-2566). Table 5 summarizes the most relevant and significant patents and patent applications associated with Trofinetide.

## 4. Discussion

**Figure 4:**
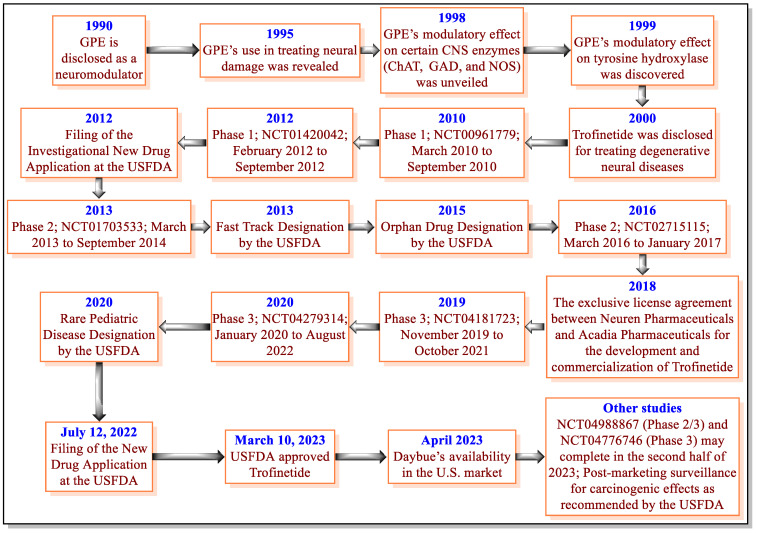
Discovery and development phases of Trofinetide.

RTT is a severe burden on patients and their family members because it affects all aspects of the quality of life of RTT patients and requires lifelong medical care and support [14,34,40]. Therefore, the USFDA approval of Trofinetide is one of the important milestones for RTT therapy. Trofinetide was discovered based on the naturally occurring neuroprotective tripeptide called GPE (Figure 2). Before the discovery of Trofinetide, GPE demonstrated modulatory effects on neurons and certain CNS enzymes (ChAT, GAD, NOS, and tyrosine hydroxylase) and was claimed to treat neurodegenerative diseases [47,48,49,50]. However, GPE demonstrated some pharmacokinetic-related concerns [69]. These observations led to the discovery of Trofinetide in 2000 by Neuren Pharmaceuticals [51]. The important milestones for the discovery and development of Trofinetide are provided in Figure 4.

GPE was derivatized to Trofinetide, wherein incorporating the α-methyl group improved the pharmacokinetic profile (half-life, oral bioavailability, and stability against enzymatic degradation) and neuroprotective activity [24,31,41,51,69]. The half-life of Trofinetide is about 1.5 h, necessitating its dosage administration twice a day (about 10 g to 24 g per day, depending on the patient’s weight) (Table 4). The modification of GPE to Trofinetide indicates possibilities of further modifications in GPE or Trofinetide to get another GPE analog, prodrug/conjugate of Trofinetide, having increased half-life, potency, and improved safety profile [70]. This development may provide once-a-day patient-compliant treatment.

IGF-1 is found in the human brain and converts to the neuro-protective tripeptide GPA [14,22,23,24,25]. IGF-1 also has a protective effect in RTT mice and patients [29,50,60,71,72]. Treating MeCP2 mutant mice with IGF-1 has improved locomotor functions, breathing patterns, heart rate irregularities, brain weight and extended life span [71]. Mecasermin (a recombinant human IGF-1) also improved the clinical features of RTT [72]. These findings suggest that IGF-1 or its analogs may be promising candidates for further research for RTT treatment.

Developing a sustained-release dosage form of Trofinetide may also be explored to get a patient-compliant treatment. Treatment of RTT requires more than one drug based on the signs and symptoms of RTT. Trofinetide is an inhibitor of OATP1B1, OATP1B3, and CYP3A4. Accordingly, drug interaction possibilities must be considered before the concomitant administration of any medicine or food item that OATP1B1, OATP1B3, and CYP3A4 metabolize. The mutations in MECP2 have been implicated in cancer development [73]. The carcinogenicity of Trofinetide has not been established yet. The USFDA has asked Acadia Pharmaceutical to assess the carcinogenicity of Trofinetide during the post-marketing surveillance [35,36]. The data of this post-surveillance study will be useful in decision making for RTT patients who have cancer. Trofinetide is water soluble and is mainly excreted in the urine, indicating the kidney is the major organ involved in its excretion from the body (Table 4). This necessitates Trofinetide monitoring among geriatric patients and patients with compromised kidney functions.

**Figure 5:**
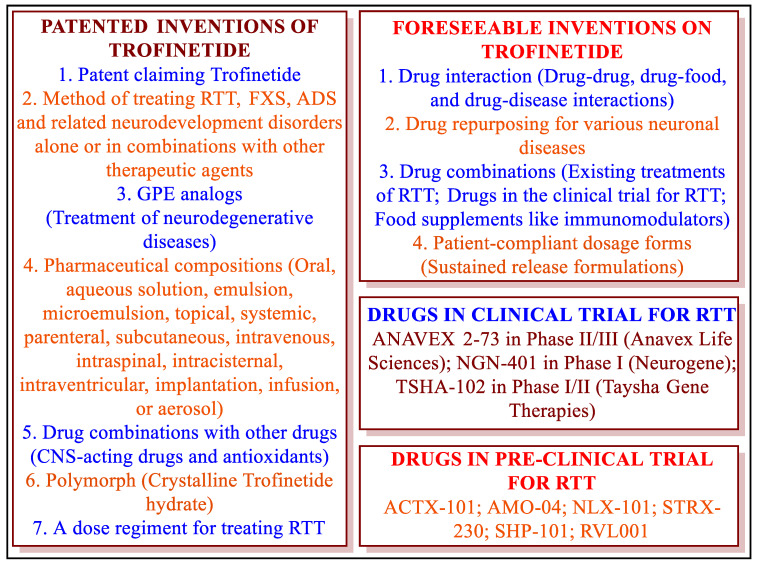
Inventions on Trofinetide and drugs of the future for RTT.

The patent literature on a drug provides information about the inventions and innovations related to a drug. This information helps develop other drug inventions [74,75]. Our patent literature search provided different inventions related to Trofinetide (Table 5). The compositions of Trofinetides have been claimed with many CNS-acting drugs for treating RTT, FXS, and brain injuries. However, the patents claiming these compositions are silent about the experimental evidence of such claims. Similarly, different compositions of Trofinetide for administration by different routes have been suggested without experimental evidence. The treatment and invention-related knowledge gaps discussed above allow scientists to investigate the practical effects of unexplored compositions and new treatments to benefit patients (Figure 5).

A mutation in the MECP2 gene causes RTT. The exact mutation in the MECP2 gene responsible for RTT is unclear [12]. A clear understanding of the exact mutation responsible for RTT will help to develop precision medicine for RTT. Utilizing anti-inflammatory drugs during brain injury is well known [31]. Trofinetide enhances antioxidant responses in the brain [8]. Therefore, the authors recommend assessing the effects of the combinations of Trofinetide with known antioxidants and NSAIDs (such as cyclooxygenase-2 inhibitors) for treating RTT. Trofinetide is also assessed for other neuronal diseases such as FXS (NCT01894958; Phase II completed), traumatic brain injuries (NCT01420042 and NCT00961779), and concussion (NCT02100150; Phase II terminated) [25,33,39]. Being an analog of GPE, Trofinetide can show promising results for certain CNS diseases (Huntington’s disease, Parkinson’s disease, and Alzheimer’s disease). It is expected that Trofinetide may be approved for treating FXS and other neuronal degenerative diseases. Interestingly, many new therapies are also in clinical and pre-clinical trials for RTT (Figure 5). The literature has also cited some innovative treatments for RTT (gene therapy, gene replacement strategies, genome editing, RNA editing, and reactivation of the inactive x chromosome) [1,15]. Accordingly, the authors consider that the USFDA approval of Trofinetide is the beginning of a new era for treating RTT, FXS, ASD, brain injury, stroke, and other neurodevelopmental disorders [24].

## 5. Conclusions

Trofinetide’s recent USFDA approval marks a significant step forward in treating RTT. Patients and their families affected by RTT would benefit greatly from this approval since they have had few alternatives for alleviating the wide range of symptoms that this condition can bring. The approval of Trofinetide will also speed up the development of new treatments and inventions for RTT and related disorders (FXS, ASD, brain injury, and stroke). Innovative Trofinetide-based molecules, patient-compliant formulations, precise MECP2-mutation-related personalized medicines, and new combinations of Trofinetide with existing RTT-based treatments, CNS-acting drugs, NSAIDs, and immunomodulators (antioxidants) are all likely shortly.

## Figures and Tables

**Table 1:** Rx data of Trofinetide.

Active Ingredient (Proprietary Name; Applicant)	Dosage Form (Route; Strength; Approval Date; Marketing Status)	Marketing Exclusivity	Approved Indication
Trofinetide (Daybue; Acadia Pharmaceuticals)	Solution (Oral; 200 mg/mL; 10 March 2023; Prescription)	New chemical entity exclusivity expires on 10 March 2028; Orphan drug exclusivity expires on 10 March 2030	Rett syndrome or symptoms thereof in patients of ≥2 years of age

**Table 2:** Summary of important non-clinical study parameters of Trofinetide [36,37].

Parameter	Finding
**Mechanism of action (MoA)**	There was no specific description of the MoA. According to the sponsor, Trofinetide improves neuronal and synaptic functioning and morphology.
**Kinase binding assay**	No significant evidence of strong binding/inhibition was found.
**Safety**	No adverse effects in rats [CNS (350 mg/kg, IV bolus); respiratory system (700 mg/kg, IV single dose); heart rate or arterial blood pressure (800 mg/kg, IV single dose)]; the QTcV interval was slightly prolonged at dose ≥ 400 mg/kg; IC_50_ (≥20 mM) on hERG current.
**ADME**	T_max_ of approximately 2 h (oral 60, 90, 600, 600, and 900 mg/kg/day) in rats; distributed to most tissues in rats (oral 200 mg/kg); no metabolite was revealed brain, plasma, urine feces, or bile of rat (oral 200 mg/kg); excreted in the urine and feces.
**Toxicology**	Well tolerated (1000 mg/kg BID, oral, 26 weeks) in rats and (500 mg/kg BID, oral, 39 weeks) in dogs.
**Genetic toxicology**	No mutagenic effect in the Ames assay (2000 mg/kg/day, oral); negative results in genetic-related assays.
**Fertility**	No adverse effects in female or male rats (1000 mg/kg BID, oral).
**Carcinogenicity**	No study has been conducted yet. The USFDA has asked to conduct a carcinogenicity study during postmarketing surveillance.
**Pre- and postnatal development**	None of the doses tested (up to 1000 mg/kg BID, oral) caused any ill effects on pregnant rats.
**Embryofetal development studies**	No adverse effects were observed in rats (up to 1000 mg/kg BID, oral) and rabbits (up to 300 mg/kg BID, oral) during the period of organogenesis.

**Table 3:** Interventional clinical studies on Trofinetide for the treatment of RTT [39].

NCT Number (Other IDs; Title Acronym; Sponsor; Locations)	Phase (Status; Numbers Enrolled; Results; Last Update on Database; Completion Date)	Primary Purpose (Intervention)
**NCT04181723** (ACP-2566-003; Lavender; Acadia Pharmaceuticals; United States)	3 (Completed; 187; Submitted, but not published; 2 November 2022; 28 October 2021)	Treatment (Efficacy of Trofinetide solution (oral or by gastrostomy tube) versus placebo in females (5–20 years) with RTT)
**NCT04279314** (ACP-2566-004; LILAC™; Acadia Pharmaceuticals; United States)	3 (Completed; 154; No result posted; 23 September 2022; 19 August 2022)	Treatment (Safety and tolerability of Trofinetide (oral or by gastrostomy tube) in girls and women with RTT for 40 weeks)
**NCT04776746** (ACP-2566-005; Not mentioned; Acadia Pharmaceuticals; United States)	3 (Active, not recruiting; 78; No result posted; 4 April 2023; July 2023)	Treatment (Safety and tolerability of Trofinetide (oral or by gastrostomy tube) in girls and women with RTT for 32 months)
**NCT04988867** (ACP-2566-009; Daffodil™; Acadia Pharmaceuticals; United States)	2/3 (Active, not recruiting; 15; No result posted; 6 May 2022; July 2023)	Treatment (Safety and tolerability of oral solution of Trofinetide in girls of 2–5 years age suffering from RTT)
**NCT02715115** (Neu-2566-RETT-002; Not mentioned; Acadia Pharmaceuticals; United States)	2 (Completed; 82; Published; 14 August 2020; 5 January 2017)	Treatment (Safety and tolerability of Trofinetide solution in females (5–15 years) with RTT)
**NCT01703533** (Neu-2566-RETT-001; Not mentioned; Acadia Pharmaceuticals; United States)	2 (Completed; 67; No result posted; 5 February 2018; September 2014)	Treatment (Safety and tolerability of Trofinetide solution (35 to 70 mg/kg BID) in adolescent/adult females with RTT)
**NCT00961779** (Neu-2566-HV-004; Not mentioned; Neuren Pharmaceuticals; Australia)	1 (Completed; 39; No result posted; 7 October 2014; September 2010)	Treatment (Safety and pharmacokinetic parameters of NNZ-2566 in healthy female)
**NCT01420042** (Neu-2566-HV-005; Not mentioned; Neuren Pharmaceuticals; Australia)	1 (Completed; 24; No result posted; 22 November 2012; September 2012)	Treatment (Safety and pharmacokinetic parameters of NNZ-2566)

**Table 4:** Pharmacological parameters of Trofinetide.

Parameter	Summary
**Dosage form; Route**	Pink-red strawberry-flavored solution (200 mg/mL) in 500 mL capacity bottle containing 450 mL solution; Oral and via gastrostomy (G) tube
**Dose**	Two times a day (BD) (morning and evening) based on the patient’s weight (25 mL BD, 9 kg to <12 kg; 30 mL BD, ≥12 kg < 20 kg; 40 mL BD, ≥20 kg < 35 kg; 50 mL BD, ≥35 kg < 50 kg; 60 mL BD, ≥50 kg); Trofinetide can be taken with or without food
**Pharmacokinetic parameters**	C_max_ = 139.5–215.8 μg/mL; T_max_ = 2–3 h; AUC = 839.6–1109.2 ng/mL/hour; Half-life = 1.5 h; Bioavailability = 84%; Distribution = 80 L; Protein binding ≤ 6%; Excreted unchanged (up to 80%) in urine with minor excretion in feces (15%); No information about clearance
**Metabolism**	CYP450 and uridine diphosphate glucuronosyltransferase (UGT) play a non-significant role in Trofinetide metabolism; Hepatic metabolism is non-significant; No major metabolites are reported; Trofinetide is a weak inhibitor of CYP3A4; In vitro, studies revealed that Trofinetide is an inhibitor of OATP1B1 and OATP1B3.
**Adverse reaction**	Mild to moderate Diarrhea (up to 85% of patients), weight loss (decreased appetite), vomiting, fever, seizure, anxiety, fatigue, and nasopharyngitis. The concomitant administration of loperamide can handle diarrhea.
**Warning**	Diarrohea is one of the major adverse effects. Therefore, concomitant laxative use must be avoided; Weight loss monitoring is also required.
**Drug/food interaction**	No significant effect is expected with co-administration of CYP450 enzyme inducers or inhibitors; Trofinetide can increase the AUC of drugs metabolized by CYP3A4 (e.g., midazolam); Drug monitoring is needed with concomitant use of drugs metabolized via OATP1B1 and OATP1B3; High-fat meal affects the absorption of Trofinetide, but to a negligible extent; No contraindication is revealed for Trofinetide.
**Special population**	Pregnancy and lactation (No adequate data generated as pregnancy among RTT patients is rarely expected); Geriatric patients and patients with compromised kidney/liver functions need drug monitoring due to lack of proper safety data.
**Toxicity**	No information about acute toxicity (LD_50_)
**QT prolongation**	Trofinetide can prolong QTcF interval; No significant effect on ECG.

**Table 5:** Relevant patents of Trofinetide.

Patent/Application Number (Status on 6 May 2023; Applicant; Filing Date; Publication Date)	Summary
**EP0366638A2** (Withdrawn; Kabigen; 24 October 1989; 2 May 1990)	This patent application disclosed that some peptides, including GPE (gly-pro-glu), are effective as a neuromodulator. These peptides either stimulate or inhibit neural activity within the CNS and affect the electrical properties of neurons. It claimed the use of GPE for medicinal and diagnostic use [47].
**WO9517204A1** (Lapsed; Auckland Uniservices Limited; 20 December 1994; 29 June 1995)	This patent application revealed GPE’s neuroprotective effects. It claimed a method of treating neural damage with a therapeutically effective amount of GPE [48].
**WO9814202A1** (Lapsed; Auckland Uniservices Limited; 6 October 1997; 9 April 1998)	This patent application unveiled that administration of GPE augments the concentration of choline acetyltransferase (ChAT), glutamic acid decarboxylase (GAD), and nitric oxide synthase (NOS) in CNS. It claimed a method of treating diseases caused by the enzymes’ imbalanced activity [49].
**WO9965509A1** (Lapsed; Neuronz Limited; 15 June 1999; 23 December 1999)	This patent application discovered that GPE’s administration could augment the concentration of tyrosine hydroxylase in the brain and may be useful for treating Parkinson’s disease [50].
**US7304029B1** (Expired; Neuren Pharmaceuticals; 3 September 1999; 4 December 2007)	A method of protecting neurons from degeneration, ischemia, or hypoxia by administering growth hormone or its derivatives, including GPE and IGF-1 [51].
**WO0216408A2** (Withdrawn; Neuronz Limited; 24 August 2001; 28 February 2002)	This patent application disclosed GPE analogs for treating brain injury and neurodegenerative diseases [52].
**US7041314B2** (Expired; Neuren Pharmaceuticals; 24 May 2002; 9 May 2006)	This patent generically claims Trofinetide and its pharmaceutical compositions with a pharmaceutically acceptable excipient for administration by different routes (intravenous, subcutaneous, topical, intraspinal, aerosol, etc.). It further claims treatment of neural degeneration by administering the claimed compositions of Trofinetide [53].
**US7605177B2** (Patented case; Neuren Pharmaceuticals; 20 December 2005; 20 October 2009)	Treating neurological injury caused by traumatic brain injury by administering a therapeutically effective amount of Trofinetide alone or in combination with another anti-apoptotic or neuroprotective agent. It also claims treatment of seizures induced by traumatic brain injury by administering a pharmacologically effective amount of Trofinetide [54].
**US7714020B2** (Patented case; Neuren Pharmaceuticals; 4 April 2006; 11 May 2010)	A treatment of traumatic brain injury and non-convulsive seizure using Trofinetide (0.01–10 mg/kg/hour) alone or in combination with another therapeutic agent (phenytoin, phenobarbital, diazepam, acetazolamide, dextromethorphan, etc.) [55].
**US7887839B2** (Patented case; Neuren Pharmaceuticals; 15 September 2008; 15 February 2011)	A pharmaceutical emulsion comprising an oil, a surfactant (polyoxyethylene (20) sorbitan monooleate, and sorbitan monooleate), and Trofinetide. The composition may optionally contain a permeability enhancer (sodium caprate and sodium taurocholate). It also claims a tablet comprising the above-mentioned pharmaceutical composition, a binder, and an enteric coating. The claimed composition can be used for treating neurological disorders [56].
**US8178125B2** (Patented case; Neuren Pharmaceuticals; 14 February 2011; 15 May 2012)	A pharmaceutical emulsion composition comprising one caprylic triglyceride, a surfactant, and Trofinetide [57].
**US8496963B2** (Patented case; Neuren Pharmaceuticals; 14 May 2012; 30 July 2013)	An emulsion comprising Trofinetide and a lipid-containing carboxylic acid. It also claims pharmaceutical composition comprising Trofinetide and a peptide conjugating agent (ethyl 2-cyanoacrylate) [58].
**US9708366B2** (Patented case; Neuren Pharmaceuticals; 27 January 2012; 18 July 2017)	A method for treating Fragile X Syndrome (FXS) using an aqueous solution of Trofinetide (0.001 to 100 mg/Kg) alone or combined with a second therapeutic agent [59].
**US2015224164A1** (Discontinued; Neuren Pharmaceuticals; 9 February 2015; 13 August 2015)	A method for treating autism spectrum disorder (RTT, FXS, etc.) using Trofinetide alone or combined with another therapeutic agent on a need basis [60].
**US9212204B2** (Patented case; Neuren Pharmaceuticals; 26 January 2015; 15 December 2015)	This patent claims a method for treating RTT symptoms with an aqueous solution of Trofinetide. The claimed solution may contain a second therapeutic agent, including IGF-I, GPE, selegiline, and fluoxetine [61].
**US11612642B2** (Patented case; Beyond Barriers Therapeutics; 27 April 2020; 28 March 2023)	A method of treating a CNS disorder using an antioxidant (N-acetylcysteine to increase glutathione level in the brain) intranasally alone or in combination with Trofinetide, progesterone, matrix metallopeptidase 9, or NSAIDs [62].
**US11370755B2** (Patented case; Neuren Pharmaceuticals; 14 June 2021; 28 June 2022)	The patent relates to the compositions and commercially feasible manufacturing process of Trofinetide. It claims a composition comprising Trofinetide and a side product of Formula II (0.001 to 2 wt%) produced during the manufacturing process of Trofinetide. It also claims a composition comprising about 98 wt% and 100 wt% of Trofinetide on an anhydrous basis [63].
**US2022324799A1** (Under examination; Neuren Pharmaceuticals; 23 June 2022; 13 October 2022)	The compositions and commercially feasible manufacturing process of Trofinetide. A composition comprising Trofinetide and two side products (Formula II or Formula III in a concentration of 0.001 to 2 wt%) produced during the manufacturing process of Trofinetide. It also claims a kit containing a dosage form comprising Trofinetide, a side product of Formula II (0.001 to 2 wt%) produced during the manufacturing process of Trofinetide, and instructions for administration to a subject in need thereof [64].
**US2023023114A1** (Under examination; Acadia Pharmaceuticals; 12 July 2022; 26 January 2023)	Crystalline Trofinetide hydrate is characterized by its powder x-ray diffraction pattern, d-spacings, FT-Raman spectrum, low-frequency Raman spectrum, 13C solid-state nuclear magnetic resonance spectrum, melting point (71.71–72.06 °C, infrared spectrum, near-infrared spectrum or a combination thereof. It also claims an aqueous pharmaceutical formulation comprising the crystalline Trofinetide hydrate for treating RTT, FXS, autism spectrum disorder, and other neurodevelopmental disorders [65].
**US2022339138A1** (Under examination; Acadia Pharmaceuticals; 1 February 2022; 27 October 2022)	A method of treating RTT by administering a pharmaceutical composition of Trofinetide to the patient with MECP2 mutation in a daily amount of (a) 4–10 g if the patient weighs between 8 and 11.9 kg; (b) 10.1–14.0 g if the patient weighs between 12–20 kg; (c) 14.1–18.0 g if the patient weighs between 20.1 and 35 kg; (d) 18.1–22.0 g if the patient weighs between 35.1 and 50 kg; or (e) 22.1–26 g if the patient weighs between 50.1 and 150 kg [66].
**WO2022246277A2** (No national phase entry; Harvard College and Tufts Medical Center; 20 May 2022; 24 November 2022)	A method of treating RTT and a symptom of CDKL5 deficiency disorder with a therapeutically effective amount of vorinostat, ivermectin, and Bacteroides fragilis or a polysaccharide isolated from Bacteroides fragilis alone or in combination thereof. It further claims a composition comprising (a) two or more of vorinostat, ivermectin, Trofinetide, and Bacteroides fragilis or a polysaccharide isolated from Bacteroides fragilis and (b) a pharmaceutically acceptable excipient [67].
**WO2022165250A1** (No national phase entry; University of Florida; 28 January 2022; 4 August 2022)	A method of treating a DYRK1A-related disorder (autism spectrum disorder, intellectual disability, microcephaly, and sociability deficits) using IGF-1, Trofinetide, or NNZ-2591. The claimed treatment method improves sociability, decreases microcephaly, increases spine density, and/or improves synaptic function [68].

## Data Availability

The contents of this review have been obtained from the references cited in this review.
